# Physical Activity in Functionally Monocular Persons in the United States, 2003–2006

**DOI:** 10.1167/tvst.12.2.13

**Published:** 2023-02-09

**Authors:** Chandana Papudesu, Jeffrey Ryuta Willis, Pradeep Ramulu, Suzanne van Landingham

**Affiliations:** 1 Department of Ophthalmology, University of Wisconsin-Madison, Madison, WI, USA; 2 Genentech, Inc., South San Francisco, CA, USA; 3 Wilmer Eye Institute, Johns Hopkins University, Baltimore, MD, USA

**Keywords:** low vision, monocular vision, physical activity, epidemiology, binocular vision

## Abstract

**Purpose:**

Real-world physical activity patterns in monocular persons have not been previously characterized. This study uses a nationally representative sample to compare the physical activity levels of functionally monocular to binocularly sighted persons in the United States.

**Methods:**

This cross-sectional study uses data from the 2003–2004 and 2005–2006 National Health and Nutrition Examination Survey (NHANES) to compare differences in physical activity between functionally monocular and binocular participants. The main outcome measures were accelerometer-measured mean steps per day and mean daily minutes of moderate or vigorous physical activity (MVPA). Statistical analysis was conducted using multivariable negative binomial regression models adjusted for age.

**Results:**

In total, 7967 NHANES participants had complete visual acuity and accelerometer data. The mean age at baseline was 44.5 years, and a majority were Caucasian (73%) and female (51%). In models adjusted for age only, functionally monocular participants (*n* = 172) took fewer steps (9277 with 95% confidence interval [CI], 8800–9753 vs. 10,057 with 95% CI, 9832–10,281) and engaged in similar minutes of MVPA (26.75 with 95% CI, 22.0–31.5 vs. 26.70 with 95% CI, 25.6–27.7) per day compared to binocularly sighted participants (*n* = 7758). In our final model, functionally monocular participants took 16% fewer steps per day (*P* < 0.01) and engaged in 26% fewer minutes per day of MVPA (*P* = 0.01). Poorer visual acuity, older age, female gender, obesity, congestive heart failure, and arthritis were also associated with a statistically significant decrease in physical activity in both models.

**Conclusions:**

Functionally monocular persons have lower physical activity levels compared to those with binocular eyesight in the United States, even after adjusting for better-eye visual acuity.

**Translational Relevance:**

Our translational study provides insight into the epidemiology of physical activity and its impact on population health. We quantify real-world physical activity in two at-risk populations, monocular and blind individuals.

## Introduction

Loss of binocular vision results in impaired stereopsis and peripheral visual fields, which may impact physical function. Monocular vision can be acquired from various eye diseases, including age-related macular degeneration, glaucoma, optic neuropathies, diabetic retinopathy, intraocular tumors, and trauma. Monocular individuals can experience difficulties in carrying out daily activities of living requiring hand–eye coordination and depth perception, such as cooking, crossing the street, navigating steps or curbs, playing sports, and driving.^1^^–^^4^ These individuals have also been reported to have decreased postural stability and increased risk of injurious falls and motor vehicle collisions.^5^^–^^11^ Many monocular persons can compensate for their visual limitation quite well, however, and their ability to compensate for lack of binocular vision may depend on the cause of vision loss.

Activity restriction in persons with vision loss has been widely reported.^11^^–^^14^ A nationally representative study showed that accelerometer-measured physical activity (PA) is significantly less in persons with uncorrectable vision loss compared to those with uncorrected refractive error or normal sight and that the impact of visual impairment on PA was equal to or exceeded that associated with self-reported systemic comorbidities, including chronic obstructive pulmonary disease, diabetes mellites, arthritis, stroke, or congestive heart failure.^15^ Low physical activity has also been associated with vision loss from specific eye diseases, including glaucoma, diabetic retinopathy, and age-related macular degeneration.^16^^–^^18^ Decreased PA has been associated with poor quality of life and higher morbidity and mortality,^1^^,^^12^ emphasizing the importance of studying the impact of vision impairment on physical activity.

In this study, real-world physical activity measured by accelerometer wear as part of the National Health and Nutrition Examination Survey (NHANES) was compared between functionally monocular and binocularly sighted participants, where *functionally monocular* was defined as having vision of 20/80 or better in one eye and 20/200 or worse in the other eye, or having only one eye.^19^ NHANES has been used to study the impact of vision on physical activity in the past, identifying a relationship between visual acuity and physical activity, visual field and physical activity, and diabetic retinopathy and low physical activity.^8^^,^^15^^,^^17^^,^^18^ Here, we use this nationally representative data set to test the hypothesis that monocular vision may lead to decreased physical activity.

## Materials and Methods

The NHANES 2003–2004/2005–2006 protocols were reviewed and approved by the National Center for Health Statistics research ethics review board. Informed consent was obtained from all participants. This work was Health Insurance Portability and Accountability Act compliant and adhered to the tenets of the Declaration of Helsinki.

### Study Population

The NHANES is an ongoing cross-sectional survey administered by the US Centers for Disease Control and Prevention (CDC). It is designed to reflect the health and nutritional status of the US noninstitutionalized civilian population, using a complex survey design and selective oversampling of older adults, certain ethnic minorities, and other groups.^1^ The survey comprises interviews and physical examinations performed in specifically equipped mobile centers. The current study uses data from the 2003–2004 and 2005–2006 NHANES cycles, as these are the two cycles that included both visual acuity testing and accelerometer-measured physical activity.

### Evaluation of Visual Acuity

NHANES participants underwent eye examinations at mobile eye centers. Individuals 12 years old or older underwent automated distance visual acuity and refraction measurements as previously detailed.^20^ In brief, presenting visual acuity was assessed with the participant's usual distance vision correction for each eye using the ARK-760 (Nidek Co. Ltd., Gamagori, Japan), an auto-refractor containing built-in visual acuity charts with 20/20, 20/25, 20/30, 20/40, 20/50, 20/60, 20/80, and 20/200 lines. After presenting visual acuity was measured, corrective lenses were removed (if applicable), and an automated refraction was performed for each eye with presenting visual acuity worse than 20/25. Corrected visual acuity was then measured for these eyes using the autorefractor-determined correction.

For this study, corrected visual acuity was defined as the better of visual acuity determined by autorefraction or presenting visual acuity. Corrected visual acuity was used for analyses because visual impairment has been shown to have a greater impact on physical activity than uncorrected refractive error.^15^ Visual impairment can result at birth or be acquired later in life. Based on these results, participants’ visual acuity was classified as functionally binocular or functionally monocular. Patients with visual acuity better than 20/200 bilaterally had their visual acuity classified as functionally binocular. Functionally monocular participants had a visual acuity better than 20/200 in one eye and 20/200 or worse in the other eye. Participants who wore an eyepatch or prosthesis on one side were classified as functionally monocular. Monocular participants were divided into those with vision of 20/40 or better and those with vision between 20/200 and 20/40 in their better-seeing eye in order to explore if physical activity was affected by the level of vision loss in the monocular population. Participants were excluded if they had missing visual acuity data in both eyes, were functionally blind, or had a severe eye infection in one or both eyes.

### Evaluation of Physical Activity

Physical activity was measured as part of NHANES by having participants wear an accelerometer (model 7164; Actigraph, LLC, Pensacola, FL, USA) over the right hip on an elastic belt during all waking hours for 1 week, as previously described.^21^ Participants were instructed to remove the device when bathing or swimming, as the device was not waterproof. Participants were excluded from physical activity monitoring if their waist size was too large to accommodate the belt, they were in a wheelchair, or they had undergone recent abdominal surgery. The device is programmed to detect and record the magnitude of linear acceleration produced by locomotion or other activity over successive 1-minute intervals. After the 7 days, the device was returned to download the data and ensure it was calibrated to the manufacturer's specification. If the device did not meet calibration criteria upon return, then the data were excluded from analysis.

Ambulatory activity was measured as counts per minute and steps per minute. The raw accelerometer data were processed using a publicly available code.^22^ The intensity of activity was characterized in each 1-minute interval as sedentary/light activity with 0 to 2019 counts or moderate/vigorous physical activity (MVPA) level with counts of 2020 or more.^22^ Participants with invalid data were excluded from the analysis. Data were considered invalid if the participant had fewer than 4 days of accelerometer wear, defined as 10 or more hours of wear, or the device was inappropriately calibrated upon return.

### Evaluation of Covariables

Interviews were conducted as part of NHANES to obtain basic demographic and medical information, including age, gender, ethnicity, education level, and medical comorbidities.^20^^,^^21^ Ethnicity was self-reported as non-Hispanic white, non-Hispanic black, Mexican American, other Hispanic, or other. Education level was self-reported and characterized as any college versus no college. Medical history was gathered by asking participants, “Has a doctor or other health professional ever told you that you had [specific condition]?” Data about obesity, diabetes mellitus, stroke, congestive heart failure (CHF), arthritis, chronic obstructive pulmonary disease (COPD), and asthma were evaluated in this study, as these conditions have been shown to affect physical activity.^13^^,^^22^

Balance was assessed in the 2003–2004 cycle using a questionnaire as well as a modified Romberg Test of Standing Balance on Firm and Compliant Support Surfaces for adults ages 40 and older.^23^ The questionnaire asked participants, “During the past 12 months, have you had dizziness, difficulty with balance, or difficulty with falling?” Participants answering “yes” were then asked specifically about dizziness, balance, and falling. The modified Romberg test evaluates participant steadiness under four increasingly challenging conditions that diminish vestibular, vision, and/or proprioceptive input. Test failure was declared when a participant opened their eyes in an eyes-closed test condition, moved their arms or feet in order to achieve stability, began to fall, or required physical support from an examination technician for any of the four conditions.

### Statistical Analysis

The NHANES survey used a complex, multistage, probability sampling design to select participants representative of the civilian, noninstitutionalized US population.^24^ Publicly available sample weights computed by the National Center for Health Statistics were therefore used in all analyses. Differences in demographic and health characteristics between the functionally binocular and functionally monocular groups were compared using Wald and χ^2^ testing.

The relationship between physical activity and monocular vision was assessed using univariable and multivariable negative binomial regression models. Negative binomial regression models were used because the accelerometer-generated physical activity count data showed evidence of overdispersion. All models were adjusted for age given the strong relationship between age and physical activity and the age differences between our study groups. The final model also included gender, ethnicity, education level, better eye visual acuity, and systemic health conditions (obesity, diabetes mellitus, stroke, CHF, arthritis, COPD, and asthma) as covariables. Summary measures of physical activity were generated in SAS version 9.2 (SAS Institute, Inc., Cary, NC, USA), all other analysis was performed in Stata version 16.1 (Stata Corp, College Station, TX, USA).

## Results

Of the 14,611 participants aged 12 years or older who were enrolled in the NHANES 2003–2004 and 2005–2006 cycles, 7967 had complete interview and physical examination data and were therefore eligible for this study (Table 1). The mean age of study participants at baseline was 44.5 years. Most participants were Caucasian (73.4%), were female, (51.1%), and had some college education (53.3%). Prevalent comorbidities in the study population with complete data included obesity (30.1%; confidence interval [CI], 28.0–32.2), arthritis (25.9%; 95% CI, 24.2–27.6), COPD/asthma (10.5%; 95% CI, 9.5–11.7), and diabetes mellitus (7%; 95% CI, 6.1–8.1).

**Table 1.** Demographic and Health Characteristics of Study Participants and Ineligible NHANES Participants

Variable	Study Participants (*n* = 7967)	Ineligible NHANES Participants (*n* = 6644)	*P* Value
Better-eye visual acuity, logMAR^a^	0.05 (0.05–0.05)	0.05 (0.05–0.05)	0.6
Better-eye visual acuity, Snellen equivalent (to nearest line)^a^	20/25 (20/25–20/25)	20/25 (20/25–20/25)	
Age in years, %			<0.01
12–19	14.2 (13.0–15.5)	6.3 (4.3–9.0)	
20–29	16.4 (14.9–18.0)	13.8 (9.0–20.6)	
30–39	16.7 (15.4–17.9)	20.2 (14.8–26.9)	
40–49	18.4 (17.0–19.9)	20.8 (16.0–26.7)	
50–59	15.0 (13.4–16.6)	13.4 (9.5–18.6)	
60–69	9.3 (8.4–10.3)	9.4 (7.0–12.5)	
70+	10.1 (8.8–11.6)	16.1 (12.6–20.6)	
Gender, % male	48.8 (47.4–50.1)	41.9 (37.2–46.7)	<0.01
Race/ethnicity, %			<0.01
Non-Hispanic white	71.7 (67.1–76.0)	56.0 (45.2–66.2)	
Non-Hispanic black	11.5 (8.9–14.6)	19.7 (13.7–27.6)	
Mexican American	8.0 (6.1–10.6)	14.2 (9.3–21.0)	
Other Hispanic	3.6 (2.8–4.6)	2.6 (1.1–6.2)	
Other	5.2 (4.2–6.4)	7.5 (4.2–13.2)	
Education: Any college, %	53.0 (49.7–54.4)	43.5 (37.0–50.3)	0.02
Health, %			
Obesity	29.2 (27.1–31.3)	28.0 (22.1–34.7)	0.7
Diabetes mellitus	6.3 (5.5–7.3)	7.3 (4.7–11.3)	0.51
Stroke	2.3 (1.9–2.8)	4.3 (2.6–7.1)	<0.01
CHF	2.3 (2.9–2.8)	4.3 (2.5–7.2)	0.03
Arthritis	23.9 (22.2–25.6)	23.7 (19.2–29.0)	0.96
COPD/asthma	10.4 (9.4–11.5)	10.5 (7.8–13.9)	0.98

The ineligible group consists of NHANES participants who are 12 years old or older but do not have complete physical activity and visual acuity data. All values are proportions with 95% confidence intervals indicated in parentheses. *P* value is calculated using Pearson's χ^2^ analysis.

a Participants ineligible due to missing visual acuity (*n* = 4928) are excluded.

Compared to NHANES participants without complete interview and physical examination data, our study population was older, more likely to be male, more likely to be non-Hispanic white, and more likely to have some college education (*P* < 0.01). Our study population was significantly less likely to report a history of stroke and arthritis (*P* < 0.01 for both). No significant differences were reported between the groups for obesity, diabetes mellites, CHF, COPD and asthma.

Of 7967 study participants, 7758 had functionally binocular vision and 172 were functionally monocular. Demographic characteristics of binocular and functionally monocular groups were compared (Table 2^3^). In functionally binocular participants, corrected visual acuity (VA) of the better-seeing eye was 20/20 (95% CI, 20/20, 20/25) compared to 20/30 (95% CI, 20/25, 20/40) in functionally monocular participants (*P* < 0.01). All functionally monocular participants had a visual acuity of 20/80 or better in the better seeing eye. The median log of minimum angle of resolution (logMAR) difference between better- and worse-eye visual acuity was 0.7 (interquartile range, 0.4–0.8). Compared to binocularly sighted participants, functionally monocular participants were older, had worse vision in their sighted eye, and were less likely to have attended college. They were significantly more likely to be obese and to report a history of diabetes mellites, stroke, CHF, arthritis, and COPD or asthma (*P* < 0.01 for all). There were no significant differences in gender or race/ethnicity across visual acuity status.

**Table 2.** Characteristics of Analyzed Study Participants by Visual Acuity Status

Variable	Functionally Binocular^a^ (*n* = 7758)	Functionally Monocular^b^ (*n* = 172)	*P* Value
Better-eye visual acuity, logMAR	0.04 (0.04–0.05)	0.21 (0.14–0.27)	<0.01
Better-eye visual acuity, Snellen equivalent (to nearest line)	20/20 (20/20–20/25)	20/32 (20/25–20/40)	<0.01
Age in years, %			<0.01
12–19	11.5 (10.4–12.6)	2.0 (0.9–4.1)	<0.01
20–29	13.2 (11.9–14.5)	1.1 (0.27–4.6)	
30–39	16.4 (15.1–17.7)	9.2 (4.3–18.5)	
40–49	19.9 (18.4–21.5)	10.6 (5.2–20.3)	
50–59	17.7 (15.9–19.6)	17.2 (10.0–27.9)	
60–69	11.2 (10.2–12.3)	17.2 (12.0–24.2)	
70+	10.3 (9.0–11.7)	42.7 (33.6–52.4)	
Gender, % male	48.8 (47.5–50.1)	52.0 (43.0–60.9)	0.44
Race/ethnicity, %			0.21
Non-Hispanic white	73.3 (68.8–77.4)	76.8 (67.1–84.4)	
Non-Hispanic black	10.0 (7.8–12.8)	12.5 (7.9–19.0)	
Mexican American	8.0 (6.0–10.6)	6.7 (3.1–13.9)	
Other Hispanic	3.5 (2.7–4.5)	2.4 (1.1–5.3)	
Other	5.2 (4.2–6.4)	1.6 (0.28–8.3)	
Education: Any college, %	53.6 (51.2–56.0)	42.2 (34.7–50.1)	<0.01
Health, %			
Obesity	29.8 (27.8–32.0)	42.8 (32.6–53.7)	0.01
Diabetes mellitus	6.8 (5.9–7.9)	17.7 (12.6–24.3)	<0.01
Stroke	2.3 (1.8–2.9)	9.1 (5.3–15.0)	<0.01
CHF	2.4 (2.0–2.9)	6.5 (2.7–14.9)	0.03
Arthritis	25.5 (23.8–27.3)	39.5 (28.2–52.0)	0.01
COPD/asthma	10.4 (9.4–11.5)	18.6 (11.2–29.3)	0.02

All values are proportions with 95% confidence intervals indicated in parentheses. *P* value is calculated using Pearson's χ^2^ analysis comparing the two groups.

a Functionally binocular participants have visual acuity of better than 20/200 in both eyes.

b Functionally monocular is defined as visual acuity of better than 20/200 in one eye and 20/200 or worse in the other eye.

**Table 3.** Relationship Between Participant Demographic and Health Characteristics and Physical Activity Measures, Adjusted for Age

Variable	Interval	Steps per Day	Minutes of MVPA per Day
Monocular vision	Versus binocular	0.76 (0.65–0.88)^a^	0.67 (0.52–90)^a^
Age	10 years older	0.94 (0.93–0.95)^a^	0.79 (0.78–0.81)^a^
Better-eye visual acuity	0.1 log unit better^b^	1.58 (1.41–1.76)^a^	1.69 (1.40–2.05)^a^
Female gender	Versus male	0.87 (0.84–0.89)^a^	0.59 (0.57–0.62)^a^
Race/ethnicity	Versus non-Hispanic white		
Non-Hispanic black		0.93 (0.89–0.97)^a^	0.93 (0.87–1.0)
Mexican American		1.1 (1.0–1.1)	1.1 (1.0–1.2)^a^
Other Hispanic		1.0 (0.94–1.1)	1.1 (1.0–1.3)^a^
Other		0.94 (0.84–1.1)	0.81 (0.70–0.94)^a^
Education: Any college	Versus no college	1.0 (0.99–1.1)	1.1 (1.1–1.2)^a^
Health			
Obese	Versus not obese	0.91 (0.88–0.94)^a^	0.72 (0.68–0.76)^a^
Diabetes mellitus	Present	0.84 (0.78–0.92)^a^	0.63 (0.53–0.74)^a^
Stroke	Present	0.69 (0.59–0.83)^a^	0.56 (0.42–0.74)^a^
CHF	Present	0.70 (0.59–0.83)^a^	0.54 (0.39–0.75)^a^
Arthritis	Present	0.88 (0.85–0.92)^a^	0.74 (0.70–0.79)^a^
COPD/asthma	Present	0.90 (0.86–0.94)^a^	0.81 (0.73–0.90)^a^

Results are expressed as rate ratios (95% CI) and are derived from negative binomial regression models including age and the independent variable of interest. A rate ratio of less than 1 indicates that the characteristic is associated with decreased physical activity.

a Statistically significant.

b 0.1 log unit is equivalent to one line on the logMAR vision chart.

**Figure 1.**
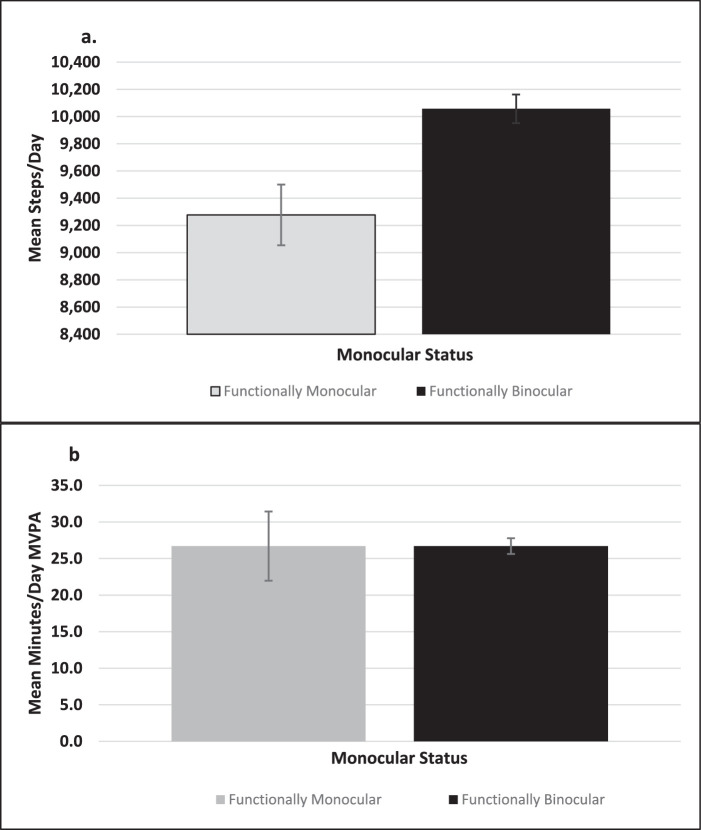
(a) Mean steps per day of functionally monocular versus binocularly sighted participants, age adjusted. (b) Mean minutes per day of MVPA of functionally monocular versus binocularly sighted participants, age adjusted. *Error bars* show 95% CI for the estimated mean steps per day versus mean minutes per day of MVPA for both figures, respectively.

On average, functionally monocular participants took fewer steps per day (7020 vs. 10,042, *P* < 0.01) and engaged in fewer minutes of MVPA (13.7 vs. 27.0, *P* < 0.01) per day compared to binocularly sighted participants. Parsed differently, 60.6% (95% CI, 52.1–68.4) of monocular participants met the American Heart Association (AHA) published goal of 10,000 steps per day compared to 73.1% (95% CI, 70.5–75.4) of binocularly sighted participants (*P* < 0.01). When adjusted for age only, functionally monocular participants took fewer steps per day (9277 with 95% CI, 8800–9753 vs. 10,057 with 95% CI, 9,832–10,281) and engaged in similar minutes of MVPA (26.75 with 95% CI, 22.0–31.5; 26.7 with 95% CI, 25.6–27.7) (Fig. 1).

Multivariable negative binomial regression models adjusting for age, better-eye visual acuity, gender, educational attainment, and health variables were built. These variables were selected based on their relationship to physical activity in univariable models and differences between vision groups. Monocular vision was associated with a 26% decrease in daily time spent in MVPA (95% CI, 16%–41) and a 16% decrease in daily steps (95% CI, 5%–26%) (Table 4). Poorer better-eye visual acuity was also strongly associated with reduced physical activity: 0.1 logMAR units worse, equivalent to one line worse on the logMAR eye chart, was associated with a 5.5% decrease in MVPA (95% CI, 3.3%–6.9%) and a 5.2% decrease in daily steps per day (95% CI, 4.0%–6.2%). Older age, female gender, obesity, CHF, and arthritis were also associated with a statistically significant decrease in PA in both models (Table 3).

**Table 4.** Multivariable Analysis of the Relationship of Monocular Vision Status and Physical Activity Measures

Variable	Interval	Steps per Day	Minutes of MVPA per Day
Monocular vision	Versus binocular	0.85 (0.76–0.95)^a^	0.74 (0.59–0.94)^a^
Age	10 years	0.95 (0.94–0.96)^a^	0.83 (0.81–0.84)^a^
Better-eye visual acuity	0.1 log unit worse^b^	0.95 (0.94–0.96)^a^	0.95 (0.93–0.97)^a^
Female gender	Versus male	0.88 (0.85–0.91)^a^	0.59 (0.56–0.63)^a^
Education: Any college	Versus no college	0.97 (0.95–0.99)^a^	1.1 (1.1–1.2)
Health			
Obese	Versus not obese	0.92 (0.89–0.94)^a^	0.74 (0.70–0.78)^a^
Diabetes mellitus	Present	0.92 (0.84–1.0)	0.75 (0.65–0.87)^a^
Stroke	Present	0.75 (0.67–0.84)^a^	0.77 (0.57–1.0)
CHF	Present	0.74 (0.62–0.87)^a^	0.64 (0.47–0.86)^a^
Arthritis	Present	0.91 (0.87–0.96)^a^	0.82 (0.76–0.89)^a^
COPD/asthma	Present	0.94 (0.90–0.99)^a^	0.91 (0.80–1.0)

Results are expressed as rate ratios (95% CI) and are derived from negative binomial regression models. A rate ratio of less than 1 indicates that the characteristic is associated with decreased physical activity.

a Statistically significant.

b 0.1 log unit is equivalent to one line on the logMAR vision chart.

Exploratory analysis was also performed for balance and fall history. Balance questionnaire data were available for 2006 of 6644 (30%) participants, and balance examination results were available for 1930 of 6644 (29%). Twenty-three of 76 (30%) functionally monocular participants and 492 of 1930 (25%) functionally binocular participants reported difficulty with dizziness, balance, or falls within the past year (age-adjusted *P* = 0.58). Differences of self-reported dizziness, balance, and falls between functionally monocular and binocular groups were also not statistically significant. When asked if their dizziness or balance problem was related to any items on a list of health variables, 2 of 23 (9%) functionally monocular and 32 of 485 (7%) functionally binocular participants selected “vision or seeing problems” (age-adjusted *P* = 0.8). Forty-five of 68 (66%) functionally monocular participants failed the modified Romberg test of balance, compared to 835 of 1732 (48%) functionally binocular participants (age- adjusted *P* = 0.5).

An additional exploratory analysis divided the functionally monocular group into persons with near-normal visual acuity in their seeing eye (vision 20/40 or better, *n* = 115) and those with more impaired visual acuity (vision between 20/200 and 20/40 in the better-seeing eye, *n* = 57). Both groups took fewer steps than binocularly sighted patients: in analyses adjusted for age only, monocular persons with near-normal vision in their better-seeing eye took 17% fewer steps (95% CI, 8–26%; *P* < 0.01) and those with reduced vision in their better-seeing eye took 40% fewer steps (95% CI, 25–52%; *P* < 0.01). In analyses adjusted for age, gender, education, and comorbidities, monocular persons with near-normal vision in their better-seeing eye took 18% fewer steps (95% CI, 4–28%; *P* = 0.02) and those with reduced vision in their better-seeing eye took 34% fewer steps (95% CI, 19–47%; *P* < 0.01). Monocular persons with impaired vision in their better-seeing eye engaged in less MVPA compared to those with binocular vision, whereas those with near-normal vision in their better-seeing eye engaged in similar amounts: in analyses adjusted for age only, monocular persons with near-normal vision in their better-seeing eye took 19% fewer steps (95% CI, −8% to 40%; *P* < 0.15), and those with reduced vision in their better-seeing eye took 62% fewer steps (95% CI, 47%–77%; *P* < 0.01). In analyses adjusted for age, gender, education, and comorbidities, monocular persons with near-normal vision in their better-seeing eye took 33% fewer steps (95% CI, −2% to 42%; *P* = 0.07) and those with reduced vision in their better-seeing eye took 56% fewer steps (95% CI, 40%–68%; *P* < 0.01).

## Discussion

Persons with monocular vision engage in less physical activity compared to those with binocular vision in this nationally representative sample of Americans. This difference was statistically significant in multivariable models controlling for age, better-eye visual acuity, gender, educational attainment, and health comorbidities. The decrease in steps per day is clinically significant: the AHA recommends that adults take 10,000 steps per day to maintain a healthy lifestyle.^25^ The binocular group achieves this goal, with mean steps per day of 10,042, while the functionally monocular group does not, with mean steps of 7020. Given the well-known associations between physical activity and general health, this suggests that functionally monocular persons may be at risk for worse cardiovascular and other health outcomes. Low-vision rehabilitation for functionally monocular persons should include strategies for safely increasing physical activity, as has been proposed by others.^2^

In a subgroup analysis, monocular persons with poorer vision in their seeing eye engaged in less physical activity than those with normal or near-normal vision in their seeing eye. This relationship is unsurprising, as a person with excellent vision in their seeing eye would be expected to have a minor visual impairment with a moderate to severe functional impairment due to lack of depth perception and reduced visual field and perhaps may be reluctant to engage in riskier physical activities only (such as contact sports), whereas a person with reduced vision in their seeing eye would be expected to be much more impaired. Similarly, in our primary multivariable models, a “dose–response” type relationship is observed between the visual acuity of the better-seeing eye and physical activity.

The relationship between physical activity and vision loss has been evaluated using real-world accelerometry data before. Previous work with NHANES data found that adult participants with bilateral visual field loss, not unilateral visual field loss, had reduced physical activity.^9^ These models adjusted for visual acuity, showing an impact of field loss on activity independent of acuity loss. Another NHANES study assessed the impact of decreased visual acuity on physical activity. These participants were characterized to have either moderate visual impairment (VA <20/40 but better than 20/200 bilaterally) or severe visual impairment (VA 20/200 or worse bilaterally). Participants in all categories of decreased VA were found to have marked restrictions in PA compared to normal-sighted participants.^2^ Accelerometry has been used to demonstrate an association of reduced physical activity with glaucomatous visual field loss^17^ and with age-related macular degeneration.^16^ Vision loss as measured by visual field and visual acuity both impact accelerometer-measured physical activity levels, with greater reductions in activity associated with poorer vision and monocular vision loss having a variable effect.

Previous studies of the relationship between monocular vision and physical activity have primarily been questionnaire based, rather than using real-world measurement techniques, and often focus on individuals who have lost an eye, omitting those who are functionally monocular.^4^^,^^26^^,^^27^ In a survey of 65 patients with a history of enucleation or evisceration surgery, 50% reported difficulties with sports and hobbies, 39% difficulty with steps and curbs, and 11% with the ability to work—activities that generally require physical activity.^4^ Kraut and Lopez-Fernandez^28^ emphasize that some sports are safer and more feasible than others for monocular persons (e.g., swimming versus baseball), and Ihrig^2^ cites a fear of bicycle riding for a patient highlighted in a case report. While these surveys indicate that a substantial number of patients with acquired monocular vision report limitations, it should be noted that many do not. In the series by Coday et al.,^4^ an overwhelming majority (89%) did not report difficulties with working. Certainly, some activities are more affected by monocular vision loss than others.

It is plausible that impaired depth perception and reduced visual field in monocular persons may contribute to concerns about safe mobility, falls, and fear of falling, which may be related to reduced activity levels. This study did not find a difference in self-reported difficulties with dizziness, balance, or falls between the functionally monocular and binocular groups, nor did it detect a difference in Romberg test failure rates. These results should be interpreted with caution, as balance data are available for only a subset of our study participants. Additionally, the accuracy of self-reported falls, particularly over a time frame of a year, is suboptimal.^29^ Furthermore, it is also possible that fear of falling may result in reduced activity independent of actual fall history.^11^^,^^30^ Identifying such mediators of reduced physical activity can help when developing targets for intervention.

Other variables associated with reduced physical activity in multivariable models include older age, lower better-eye visual acuity, female gender, and history of obesity, stroke, CHF, arthritis, and COPD/asthma. Older age is well known to be associated with reduced activity and may be in part due to age-related medical comorbidities not captured in our models.^18^ Causality in the associations between obesity, stroke, CHF, arthritis, and activity may be bidirectional. For example, stroke may be a cause of reduced physical activity (weakness due to stroke may make ambulation challenging) and/or a result of reduced physical activity (low physical activity leading to reduced cardiovascular fitness).

Strengths of our study include use of the NHANES data set, which was meticulously designed to represent the US population, and the fact that physical activity data were collected in a naturalistic fashion using accelerometers. The present study did not evaluate how long ago participants had lost their vision or how gradual their vision loss was. It was not designed to detect how functional limitations may change with time (i.e., if patients are initially more limited and then adapt to loss of eyesight) or how functional changes from different causes of vision loss (e.g., sudden loss of an eye after trauma compared to gradual loss of vision due to glaucoma) may differ. These data were collected 15 years ago, so it is possible that physical activity patterns have changed over time. An NHANES investigation showed an increase of 0.8 hours of self-reported sedentary behavior per day in the United States from 2007 to 2016, although there is no evidence to suggest that this change would have differed among those with monocular vision differently compared to those with binocular eyesight.^31^ The 2003–2004 and 2005–2006 cycles represent the last NHANES cycles in which vision and physical activity data were both collected, however, so they represent a unique opportunity to assess the relationship between these items in a nationally generalizable manner.

Accelerometers do not capture some forms of physical activity (e.g., swimming). Our data set was also underpowered to detect differences in physical activity between functionally monocular participants and truly monocular participants (those wearing an eye patch or prosthetic during the examination), although a perfunctory analysis revealed similar PA levels in both groups. Finally, the two study groups had several demographic differences: the functionally monocular group was significantly older, was less educated, and had more comorbidities compared to the binocular group. While these variables were included in the multivariable regression model to mitigate their effect on the results, it is possible that some bias remained.

In summary, monocular vision was associated with lower physical activity levels in this nationally representative sample of Americans. Persons with monocular vision loss should be encouraged to maintain a healthy level of physical activity, and rehabilitative efforts should include strategies for safely being active. More work may be helpful to elucidate the impact of timing, pace, and cause of monocular vision loss on physical activity.

## References

[1] Loprinzi PD, Sheffield J, Tyo BM, Fittipaldi-Wert J. Accelerometer-determined physical activity, mobility disability, and health. *Disabil Health J*. 2014; 7(4): 419–425. 25224982 10.1016/j.dhjo.2014.05.005

[2] Ihrig C . Vision rehabilitation team management of acquired monocular vision. *Optom Vis Sci*. 2013; 90(3): e89–e94. 23400025 10.1097/OPX.0b013e3182820d74

[3] Ihrig C, Schaefer DP. Acquired Monocular Vision Rehabilitation program. *J Rehabil Res Dev*. 2007; 44(4): 593–597. 18247256 10.1682/jrrd.2006.06.0071

[4] Coday MP, Warner MA, Jahrling KV, Rubin PA. Acquired monocular vision: Functional consequences from the patient's perspective. *Ophthalmic Plast Reconstr Surg*. 2002; 18(1): 56–63. 11910326 10.1097/00002341-200201000-00009

[5] Haymes SA, Leblanc RP, Nicolela MT, Chiasson LA, Chauhan BC. Risk of falls and motor vehicle collisions in glaucoma. *Invest Ophthalmol Vis Sci*. 2007; 48(3): 1149–1155. 17325158 10.1167/iovs.06-0886

[6] Black AA, Wood JM, Lovie-Kitchin JE, Newman BM. Visual impairment and postural sway among older adults with glaucoma. *Optom Vis Sci*. 2008; 85(6): 489–497. 18521027 10.1097/OPX.0b013e31817882db

[7] Wood JM, Lacherez P, Black AA, Cole MH, Boon MY, Kerr GK. Risk of falls, injurious falls, and other injuries resulting from visual impairment among older adults with age-related macular degeneration. *Invest Ophthalmol Vis Sci*. 2011; 52(8): 5088–5092. 21474773 10.1167/iovs.10-6644

[8] van Landingham SW, Willis JR, Vitale S, Ramulu PY. Visual field loss and accelerometer-measured physical activity in the United States. *Ophthalmology*. 2012; 119(12): 2486–2492. 22892152 10.1016/j.ophtha.2012.06.034

[9] Willis JR, Vitale SE, Agrawal Y, Ramulu PY. Visual impairment, uncorrected refractive error, and objectively measured balance in the United States. *JAMA Ophthalmol*. 2013; 131(8): 1049–1056. 23744090 10.1001/jamaophthalmol.2013.316

[10] Greguol M, Gobbi E, Carraro A. Physical activity practice among children and adolescents with visual impairment—influence of parental support and perceived barriers. *Disabil Rehabil*. 2015; 37(4): 327–330. 24828394 10.3109/09638288.2014.918194

[11] Nguyen AM, Arora KS, Swenor BK, Friedman DS, Ramulu PY. Physical activity restriction in age-related eye disease: A cross-sectional study exploring fear of falling as a potential mediator. *BMC Geriatr*. 2015; 15: 64. 26062727 10.1186/s12877-015-0062-8 PMC4464712

[12] Loprinzi PD, Joyner C. Accelerometer-determined physical activity and mortality in a national prospective cohort study: Considerations by visual acuity. *Prev Med*. 2016; 87: 18–21. 26861750 10.1016/j.ypmed.2016.02.005

[13] Troiano RP, Berrigan D, Dodd KW, Mâsse LC, Tilert T, McDowell M. Physical activity in the United States measured by accelerometer. *Med Sci Sports Exerc*. 2008; 40(1): 181–188. 18091006 10.1249/mss.0b013e31815a51b3

[14] Bajaj R, Ramulu P, Dillon L, et al. Validating the accuracy of an activity monitor in a visually impaired older population. *Ophthalmic Physiol Opt*. 2018; 38(5): 562–569. 29984414 10.1111/opo.12577

[15] Willis JR, Jefferys JL, Vitale S, Ramulu PY. Visual impairment, uncorrected refractive error, and accelerometer-defined physical activity in the United States. *Arch Ophthalmol*. 2012; 130(3): 329–335. 22411662 10.1001/archopthalmol.2011.1773

[16] Sengupta S, Nguyen AM, van Landingham SW, et al. Evaluation of real-world mobility in age-related macular degeneration. *BMC Ophthalmol*. 2015; 15: 9. 25636376 10.1186/1471-2415-15-9 PMC4328075

[17] Ramulu PY, Maul E, Hochberg C, Chan ES, Ferrucci L, Friedman DS. Real-world assessment of physical activity in glaucoma using an accelerometer. *Ophthalmology*. 2012; 119(6): 1159–1166. 22386950 10.1016/j.ophtha.2012.01.013 PMC3367112

[18] Loprinzi PD, Brodowicz GR, Sengupta S, Solomon SD, Ramulu PY. Accelerometer-assessed physical activity and diabetic retinopathy in the United States. *JAMA Ophthalmol*. 2014; 132(8): 1017–1019. 25124951 10.1001/jamaophthalmol.2014.402

[19] Centers for Disease Control and Prevention (CDC). National Center for Health Statistics (NCHS). National Health and Nutrition Examination Survey Data. Hyattsville, MD: U.S. Department of Health and Human Services, Centers for Disease Control and Prevention, 2003-2006.

[20] CDC/National Center for Health Statistics. *National Health and Nutrition Examination Survey: Vision Procedure Manual*. Atlanta, GA: Centers for Disease Control and Prevention; 2008.

[21] CDC/National Center for Health Statistics. *NHANES: Anthropometry and Physical Activity, Monitor Procedures Manual*. Atlanta, GA: Centers for Disease Prevention and Control; 2005.

[22] Van Domelen DR . Process accelerometer data from NHANES 2003-2006. In: *Web App for Processing NHANES Accelerometer Data*. 2018, https://vandomed.github.io/vandomed.github.io/process_nhanes_app.html. Accessed November, 2022.

[23] Yeom HA, Fleury J, Keller C. Risk factors for mobility limitation in community-dwelling older adults: A social ecological perspective. *Geriatr Nurs*. 2008; 29(2): 133–140. 18394514 10.1016/j.gerinurse.2007.07.002

[24] National Health and Nutrition Examination Survey. Balance Procedures Manual. Hyattsville, MD: U.S. Department of Health and Human Services, Centers for Disease Control and Prevention. 2001.

[25] National Center for Health Statistics. NHANES Web Tutorial, Hyattsville, MD: U.S. Department of Health and Human Services, Centers for Disease Control and Prevention. 2019.

[26] Lobelo F, Rohm Young D, Sallis R, et al. Routine assessment and promotion of physical activity in healthcare settings: A scientific statement from the American Heart Association. *Circulation*. 2018; 137(18): e495–e522. 29618598 10.1161/CIR.0000000000000559

[27] Linberg JV, Tillman WT, Allara RD. Recovery after loss of an eye. *Ophthalmic Plast Reconstr Surg*. 1988; 4(3): 135–138. 3154731 10.1097/00002341-198804030-00002

[28] Kondo T, Tillman WT, Schwartz TL, Linberg JV, Odom JV. Health-related quality of life after surgical removal of an eye. *Ophthalmic Plast Reconstr Surg*. 2013; 29(1): 51–56. 23299809 10.1097/IOP.0b013e318275b754 PMC3541504

[29] Kraut JA, Lopez-Fernandez V. Adaptation to monocular vision. *Int Ophthalmol Clin*. 2002; 42(3): 203–213. 12131596 10.1097/00004397-200207000-00021

[30] Cummings SR, Nevitt MC, Kidd S Forgetting falls. The limited accuracy of recall of falls in the elderly. *J Am Geriatr Soc*. 1988; 36(7): 613–616. 3385114 10.1111/j.1532-5415.1988.tb06155.x

[31] Wang MY, Rousseau J, Boisjoly H, et al. Activity limitation due to a fear of falling in older adults with eye disease. *Invest Ophthalmol Vis Sci*. 2012; 53(13): 7967–7972. 23132799 10.1167/iovs.12-10701

